# Effect of Model Body Type and Print Angle on the Accuracy of 3D-Printed Orthodontic Models

**DOI:** 10.3390/biomimetics9040217

**Published:** 2024-04-06

**Authors:** Stefan Lohfeld, Bryndon Belnap, Jean-Marc Retrouvey, Mary P. Walker

**Affiliations:** 1 Department of Oral and Craniofacial Sciences, School of Dentistry, University of Missouri-Kansas City, Kansas City, MO 64108, USA; belnbryn@gmail.com (B.B.); walkermp@umkc.edu (M.P.W.); 2 Department of Orthodontics and Dentofacial Orthopedics, School of Dentistry, University of Missouri-Kansas City, Kansas City, MO 64108, USA

**Keywords:** digital dentistry, 3D printing, accuracy, orthodontic models, clear aligners

## Abstract

The assortment of low-cost 3D printers for “in-practice” use, e.g., for clear aligner therapies, is ever increasing. To address concerns about the accuracy of orthodontic models produced on such printers when cost-efficient modes of 3D printing are employed, this study examined the effect of print model body type and print angulation on accuracy. Six printing-configuration groups were included: two model types (solid or hollow shell) combined with three print angles (0°, 70°, or 90°) with 10 models/group; all models were printed with 100 µm layer thickness using a digital light processing-based three-dimensional printer. Eleven selected structures and distances were measured on the printed models with a digital microscope and compared to the same measures on a digitized master model. The clinically acceptable range was set at ±0.25 mm difference from the master model for single tooth measurements (intra-tooth) and ±0.5 mm for cross-arch measurements (inter-tooth). For individual measurements across all models, 98% fell within clinical acceptability. For mean measurements within each model group, only canine height for the shell-0° model had a mean difference (−0.26 mm ± 0.03) outside the clinically acceptable range for intra-tooth measurements. Standard deviations for all intra-tooth measurements were within 0.07 mm. While none of the mean inter-tooth measurements exceeded the acceptability range, the standard deviations were larger (0.04 to 0.30 mm). The accuracy of the orthodontic models for clear aligner therapies was not impacted beyond the clinically acceptable range when altering model body type and print angulation to improve efficiency of 3D printing. These findings suggest greater flexibility of the practitioner to alter print settings to address time and cost efficiency in various clinical scenarios and still maintain clinically acceptable model accuracy.

## 1. Introduction

Additive manufacturing, now commonly called “3D printing” among many users, has extended, if not revolutionized, the way of manufacturing in various fields and disciplines. The initial term “Rapid Prototyping” was coined as the technology targeted the manufacturing industry for producing prototypes much faster than before. Since then, the technology has undergone changes and modifications to allow it to be used in increasingly specialized ways [1,2]. As the field of dentistry has undergone a substantial transformation as more dental processes are being digitized, such as digital impressions and CAD/CAM solutions, it is not surprising to now see 3D printing being employed for various applications.

When compared with subtractive manufacturing, 3D printing (additive manufacturing) has the potential to yield finer detail in situations where there are undercuts or complex geometric shapes when producing models and other items. Additive manufacturing also produces less waste, is faster, and allows for larger and more customized products. Though 3D printing still has some limitations regarding what kind of materials can be used, it is a technology that has vast implications in the field of dentistry [3,4].

While restorative dentistry utilizes 3D printing for various applications such as provisional restorations, dentures, and implant placement guides, the discipline of orthodontics has also adopted various applications of 3D printing. For example, this technology has been used to print orthodontic retainers, indirect bonding guides, as well as palatal expanders [5,6]. Perhaps the most extensive way additive manufacturing has been implemented is related to clear aligner therapy, where 3D printing of orthodontic models is an important step that has typically been done in commercial dental laboratories. It is also important to note that while more general dentists may be utilizing 3D printers in their practice related to restorative care, more general dentists are also providing clear aligner therapy-based orthodontic treatment and thus, may be utilizing practice-based printers for orthodontic-related care. As printing technology has advanced, it has been miniaturized and made available to private practices. This change in venue of fabrication has the potential to introduce more variation in the printing process, especially as the printing process can be modified by the operator to save time and money. Introducing more variability requires a more thorough evaluation of the factors that affect the accuracy of 3D printing. Inadequate accuracy will have a direct effect on the fit of the aligner and thus, the quality of treatment that the patient receives [7]. Allowable deviations from the designed dimensions differ based on the dental discipline application; fixed prosthodontics, for example, has higher precision requirements than orthodontics. While there is good evidence that the 3D printers used in orthodontics are adequately accurate for their intended applications when used according to manufacturer guidelines, the question remains whether the accuracy of 3D-printed orthodontic models is maintained when those guidelines are not followed to achieve more efficient modes of 3D printing. Consequently, factors that affect the accuracy of additive manufacturing have become a research focus.

Extensive evaluations of the accuracy of intraoral scanning, which is the first step of producing an orthodontic model, reported that digital scans accurately reproduce the intraoral structures as a digital impression [8,9]. The next potential source for inaccuracies in the process is the creation of the physical model from the digital scan. When evaluating inaccuracies of such models for an effective treatment, the clinically acceptable range of deviation from intended dimensions is more important than absolute dimensions. For orthodontic models, some studies suggest that any deviation less than ±0.5 mm is considered clinically acceptable [10,11]. However, many 3D-printed orthodontic models are used for purposes which require smaller deviations from the nominal dimensions. Specifically, for clear aligner fabrication, an absolute deviation of more than ±0.25 mm is considered clinically unacceptable [12,13], because the maximum tooth movement for each clear aligner ranges from 0.25 to 0.3 mm. If the accuracy of the aligner is outside this range, it would lack clinical efficacy and efficiency [14].

Utilizing the determined parameters of clinical acceptability, researchers are evaluating how variations in the printing process affect model accuracy. Printing technology, layer thickness, build angle, and model body type are some of the factors that impact accuracy and clinical acceptability of 3D-printed orthodontic models. Among the printing technologies, stereolithography (SLA) and digital light processing (DLP) are most commonly used for dental appliances, and both generally have been found to be sufficiently accurate to 3D print orthodontic models for clinical applications [8,11,14].

The thickness of each printed layer in an additive manufacturing process directly influences the number of layers that need to be printed for a model, and consequently, the time required to complete a print. Hence, this variable is of particular importance as it can be changed by the operator to print models faster. While adjusting the layer thickness has shown to cause some statistical variability in model dimensions, the variation still fell within the accepted range of clinical accuracy. However, the literature suggests that a 50 µm layer thickness yields a higher degree of accuracy and should be used when the highest degree of accuracy is needed [15,16].

Build angle is another variable that can be manipulated by the operator to improve efficiency. Printing orthodontic models flat on the platform reduces the height of the entire print envelop and hence, requires fewer layers, reducing time to complete the print job. At the other extreme, printing models vertically to the platform results in more layers and hence longer printing times, but more models can be fit onto the platform in the same print job. Some studies used print angles for their models that do not fit into an efficient workflow. For example, models printed at 65°, as seen in Kenning’s study [17], require support structures for the printed model. Although this angle allows for an increased number of models to be printed simultaneously (relative to models printed at 0°), it is less practical for a high-volume aligner fabrication workflow due to the extra time for removing the supports as well as the increased use of resin. Low-angle print jobs, such as 20° as used by Short et al. [12], do not allow for more models to be printed on the platform at a time. Thus, a smaller angle print job introduces the disadvantage of having supports while not providing the advantage of printing more models at a time.

The print angle was found to have an influence on the accuracy of the printed object [18,19], which needs to be considered when trying to improve print efficiency. For orthodontic models printed at various angles between 0° and 90°, it was observed in several studies that their overall accuracy was within clinical acceptance; however, models from one print angle showed significantly more single measurements outside clinical acceptance than models from the other tested angles, supporting the relationship between print angle and accuracy [12,20].

To save material and for SLA printing, to additionally save printing time, models may be printed as hollow shell models instead of solid ones. Solid orthodontic models have been shown to possess sufficient accuracy to be used for the fabrication of clear aligners when they are horseshoe-shaped and have a cross bar linking the posterior segments [8,10,11,16,21,22]. Shell models have the potential to substantially reduce the amount of resin used per model and the associated cost. Moreover, in SLA technology-based printing approaches, where a laser scans the cross section of a part line by line to cure the resin, a significantly shorter manufacturing time can result due to reduced scanning time. While increasing printing efficiency, shell models may have an increased risk of distortion compared to solid models. Two studies regarding shell thickness concluded that a 2 mm or higher shell wall thickness provides adequate dimensional accuracy and those same studies also reported that shell models can withstand the heat of the thermoforming process for aligner fabrication [17,22]. However, both these studies printed experimental shell models in ways that would increase the time for printing and pre-/post-processing. In one study, all models were printed at 65° and required several supports [17]. The other study printed flat on the print platform (0°), which required vent holes to be built into the models. This variation requires additional software and time prior to printing [22]. Furthermore, these vent holes must be filled with wax or putty before fabricating the thermoformed aligners on such a model, because otherwise, the thermoforming plastic will break as it is forced through the vent. In addition, the shell models printed flat on the platform are more prone to breakage during removal from the platform. 

The accuracy of shell models is also relevant for direct 3D printing of clear aligners, as these resemble a shell horseshoe shape model. Shell models have shown to match the accuracy of their solid counterparts [17,22]; however, this has not yet been tested when printing at angles that potentially increase print efficiency.

The aim of the present study was to determine whether 3D-printed models, manufactured on a desktop DLP printer typically used in a private practice, maintain clinically acceptable levels of accuracy when several factors to increase print efficiency are applied at the same time. More specifically, the accuracies of orthodontic models produced as a hollow shell or a solid model and at varying print angles were compared.

## 2. Materials and Methods

**Figure 1:**
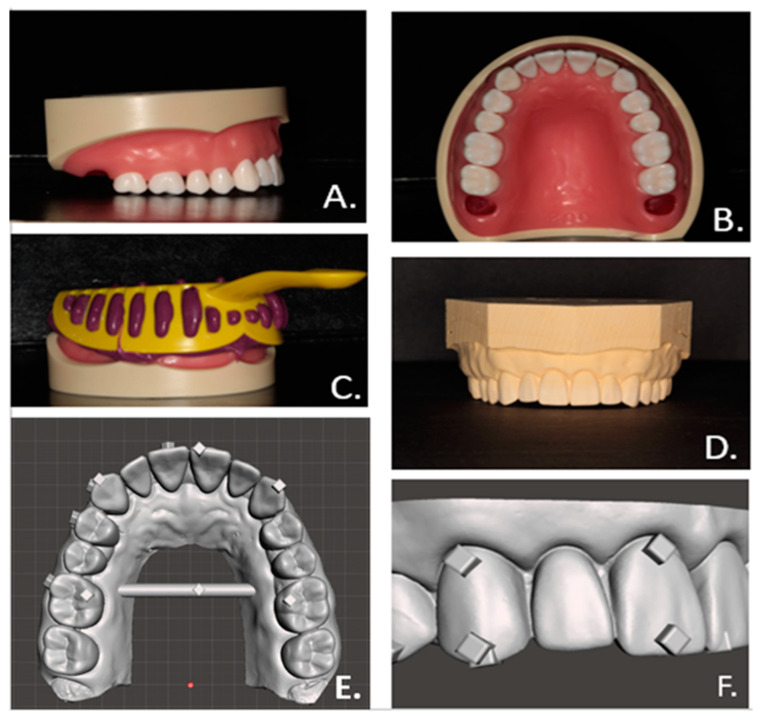
Stages of digital master model development. (**A**). Posterior buccal view of ideal maxillary typodont with 3rd molars removed. (**B**). Occlusal view of ideal maxillary typodont with 3rd molars removed. (**C**). VPS impression of typodont. (**D**). Stone model of ideal typodont. (**E**). Occlusal view of digital master model. (**F**). Zoomed in view of reference cubes on digital master model.

The master model was developed by obtaining an impression from the maxillary arch of a Kilgore NISSIN 200 Type ideal typodont (Kilgore International Inc., Coldwater, MI, USA) in vinyl polysiloxane (Aquasil Ultra Monophase, Dentsply Sirona, Charlotte, NC, USA), see Figure 1A–C. Vacuum-mixed Pemstone Golden type III stone (Garreco LLC, Heber Springs, AR, USA) was poured into the set vinyl polysiloxane (VPS) impression, allowed to set, then cleaned and trimmed (Figure 1D). Scanning of the shiny surface of the typodont model caused issues due to glare, so instead of the typodont, the stone model was scanned to create the digital master model by using an iTero Element II intraoral scanner (Align Technology, Or Yehuda, Israel). The obtained data file was uploaded into a 3D modeling software (Meshmixer, Autodesk, San Rafael, CA, USA), where a crossbar linking the posterior segments was added to ensure better arch width integrity [10] and raised 2 × 2 × 1 mm cubes were added as reference points for the accuracy evaluation measurements (Figure 1E,F).

**Figure 2:**
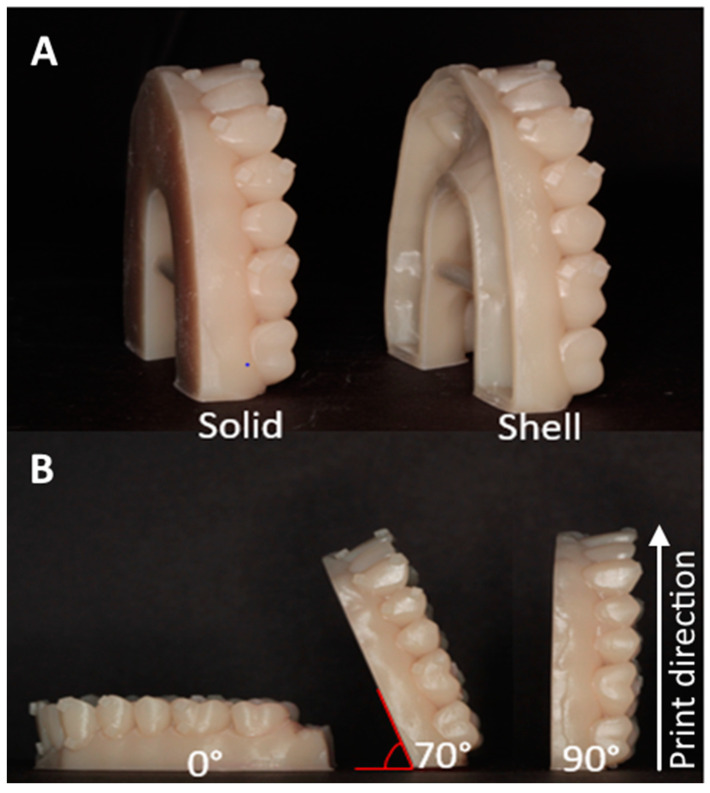
(**A**). Model body types and print angulations: solid and shell. (**B**). Three angles at which the models were printed (0°, 70°, and 90°).

Based on the digital master model file, the experimental models were printed using photosensitive polymer resin (Die and Model Tan, SprintRay, Lake Forest, CA, USA) on a calibrated DLP printer (Pro model, SprintRay) as either a solid model or as a hollow shell with shell wall thickness of 2 mm [17,22] and a print layer thickness of 100 µm. The light source has a wavelength of 405 nm. The layer exposure time for the material is a pre-set parameter for the material and layer thickness, but it is not disclosed by the manufacturer. The solid and shell experimental models (Figure 2A) were printed at the print angles 0° (horizontal), 70°, and 90° (vertical) (Figure 2B). Each print batch included one specimen of each of the six experimental group combinations of model type and print angle (solid-0°, solid-70°, solid 90°, shell-0°, shell-70°, and shell-90°). In addition, there were 10 print batches and with each print batch, the location of each model configuration was changed to minimize error from possible differences from different positions on the platform [11]. A total sample of 60 models was printed with 10 models in each experimental group. Post-processing of the models followed manufacturer recommendations with rinsing and 5 min ultrasound bath cleaning in 99% isopropyl alcohol, air drying, and post-curing for 15 min in a ProCure blue-light convection oven (SprintRay, Lake Forest, CA, USA) with a light wavelength of 405 nm.

**Figure 3:**
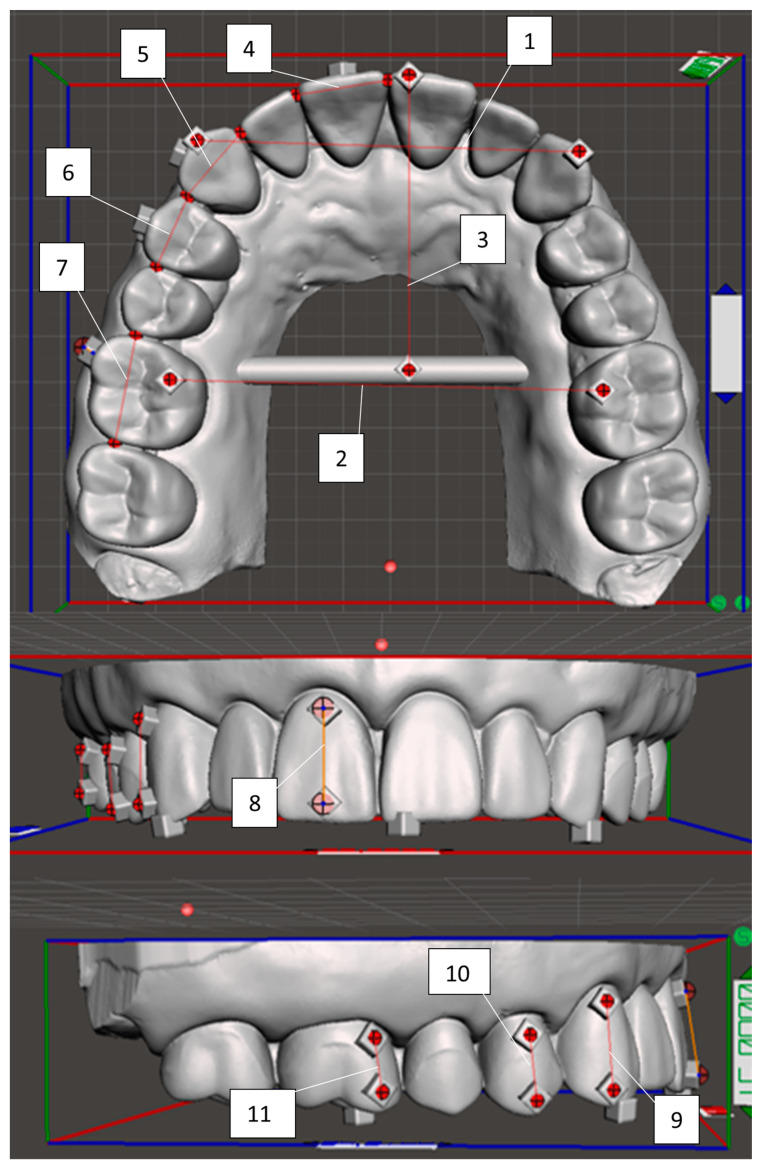
Measurements on the digital master model: 1. intercanine distance; 2. intermolar distance; 3. anteroposterior arch depth; 4. central incisor width; 5. canine width; 6. premolar width; 7. molar width; 8. central incisor height; 9. canine height; 10. premolar height; 11. molar height.

Measurements on the digital master model were performed by using the Meshmixer software. Most measurements were taken from the centers of the reference cubes added to the model, utilizing a cross hair on a transparent sheet that was overlayed on the computer screen (Supplemental Figure S1). Tooth widths, however, were measured from the interproximal contact points, as this landmark was easily and repeatedly identifiable on both the digital master model and experimental models. A total of eleven measurements were taken, as shown and defined in Figure 3. For the digital model a mean measurement was calculated for each site based on three measures per site.

Following the same measurement protocol, the experimental models were measured in randomized order by using a Measurescope MM-22 measuring microscope with 7VL stage and a level of accuracy of 0.01 mm (Nikon, Melville, NY, USA). The measuring microscope was synchronized with a monitor, a DCG-100A crosshair generator (Techniquip, Pleasanton, CA, USA), and a Quadra-Check 200 digital readout system (Medtronics Inc., Minneapolis, MN, USA). The models were stabilized with model holders made from VPS for the occlusal plane, anterior facial, and posterior buccal views to provide reproducibility of the model orientation on the measuring microscope platform (Figure S2). The platform had metal brackets superglued to the surface which served as a reference structure for the side of the jig resting on the platform. As before, the centers of the reference cubes were found utilizing the cross hair transparent sheet over the monitor (Supplemental Figure S3). Due to the digital readout system, reporting distances in the x, y, and z axes independently rather than a true total distance, Excel software (Microsoft, Redmond, WA, USA) was used to determine actual distances using the Pythagorean theorem. For each model site, three measurements were made per site and then averaged to generate the measure for that model and site. For each measure, the difference between the master model and the experimental model was calculated in mm.

All measurements of the digital master model and experimental models were performed by one investigator. Before experimental data collection began, a measurement calibration exercise was completed for the measurements on the digital master model and for randomly selected measurements across the experimental models. The same measurements were made on multiple days to assure intra-rater reliability as demonstrated by minimal variability between measurement sessions, and all repeated measurements from the three time-points of the calibration fell within a range of 0.1 mm.

Data analysis of the evaluated measures was based on comparing the mean measures from the 10 models within each experimental model group with the digital master model measures in terms of clinical acceptability. The experimental groups were solid-0°, solid-70°, solid 90°, shell-0°, shell-70°, and shell-90°. Measurement differences were defined to be clinically acceptable when not varying more than ±0.25 mm for individual tooth measurements or ±0.5 mm for cross arch measurements. In addition to mean measure comparisons, all individual measurements from across experimental group models were evaluated to determine how many data points fell outside the respective selected range of clinical acceptability.

## 3. Results

Mean differences and standard deviations of experimental models vs. the digital master model are presented in Supplemental Table S1. Of all the mean intra-tooth measurements, only one measurement fell outside the range of clinical acceptability (±0.25 mm), which was the canine height for the shell-0° model with a mean difference of −0.26 mm ± 0.03. Further, the standard deviations for all intra-tooth measurements were within 0.07 mm. None of the mean inter-tooth measurements were beyond the range of acceptability (±0.5 mm), although their standard deviations were larger and ranged from 0.04 to 0.30 mm. The number of individual measurements that fell outside the range of clinical acceptability are reported in Supplemental Table S2. Only 15 of a total of 660 recorded individual measurements had differences outside the clinically acceptable range. Thus, 98% of the individual measurements were within the selected range of clinically acceptable accuracy. For seven of the eleven measured structures and distances, all individual model measurements were clinically acceptable across 60 experimental models. The following four structures showed clinically unacceptable measurements in some cases: intermolar distance (four/60), molar crown height (one/60), canine crown height (nine/60), and central incisor crown height (one/60). The solid-70° and solid-90° models were the only models that did not have any data points outside the range of clinical acceptability. It was also noted that all 60 experimental models had crown height measurements less than the digital master model, suggesting that all the crowns were shorter, regardless of the model body type or print angle.

## 4. Discussion

With the increasing usage of 3D printing in orthodontics practices, there is increased interest in determining whether the practice-based 3D printers produce adequately accurate models, specifically as it relates to clear aligner fabrication. While most previous studies evaluated the singular effects of various printer-related factors, the present study addressed print angle and model type.

As regards the influence of the print angle on accuracy of the 3D-printed models, our investigation revealed dimensional differences depending on the print angle and model type. While for a few models statistically significant differences were detected, these were generally still within the range of clinical acceptability, which is of higher importance to the clinician for a successful treatment and hence the focus of our discussion. In comparison, the studies by Short et al. [12] and Ko et al. [20] suggest that changing the angle of the print does have a statistically significant impact on the accuracy of the models. Short et al. compared models printed on an SLA printer at the angles 0°, 20°, and 90°, where all three print angles produced models within the range of clinical acceptability. The study by Ko et al. compared models printed on a DLP technology-based printer with different layer thicknesses as well as four print angles of 0°, 30°, 60°, and 90°. Again, there were statistically significant differences; however, the average deviation from the master model for each print angle and layer thickness were within the clinically relevant range of ±0.25 mm. In summary, our study confirms previous findings that print angulation has an impact on accuracy, however, not a clinically relevant one.

In studies evaluating only the effect of model body type on model accuracy, it was determined that hollow shell models are clinically acceptable when the shell wall thickness is at least 2 mm [17,22]. In the present study, this thickness was used to test whether a shell model printed at different angles maintains clinical acceptable accuracy. Consistent with the other articles investigating model body type and accuracy, both the solid and the shell models demonstrated adequate accuracy. 

One remarkable event in Table S2 is the higher occurrence of individual measurements (seven out of 10) outside the range of clinical acceptability for the canine height for 0° shell models compared to all other measurements. The tooth height measurements are those most influenced by the layer height accuracy during printing of the 0° model prints. This is also reflected in Table S1, showing consistently higher deviations from the tooth height dimensions of the master model for both solid and shell models printed at 0°. Hence, inaccuracies in z-resolution of the printer are a possible explanation. The overall effect of this, however, is negligible, as Table S1 shows that the mean measurement was just outside the range of clinical acceptability and overall, 98% of all 660 measurements were within clinical acceptability.

Beyond evaluating single factors, only the study of Ko et al. [20] considered relationships between parameters and reported that print layer thickness and print angle have an impact on each other. The present study, however, is the first to evaluate model type in combination with print angle and to confirm there is no effect on clinical accuracy of the printed models.

### 4.1. Clinical Implications

While the goal of this study was to determine clinical acceptability of 3D-printed orthodontic models, it was also important that the models were printed in a fashion optimized for the clinical workflow. This consideration has been addressed in only a few of the previous studies. Although print angle and model body type have the potential to increase efficiency by reducing printing time and material use, when not configured properly, variations in these factors can decrease efficiency and increase cost and waste. Consequently, this study aimed to investigate printing accuracy under circumstances that would be clinically useful. Hence, it was decided to print at angles of 0°, 70°, and 90°, as none of these models require additional supports [23,24]. In addition, while the 0° shell model was included in this study for comparison with the findings by Rungrojwittayakul et al. [22], this type of shell model would not be recommended in practice as the required pre- and post-processing for making these shell models actually lower the production efficiency.

**Figure 4:**
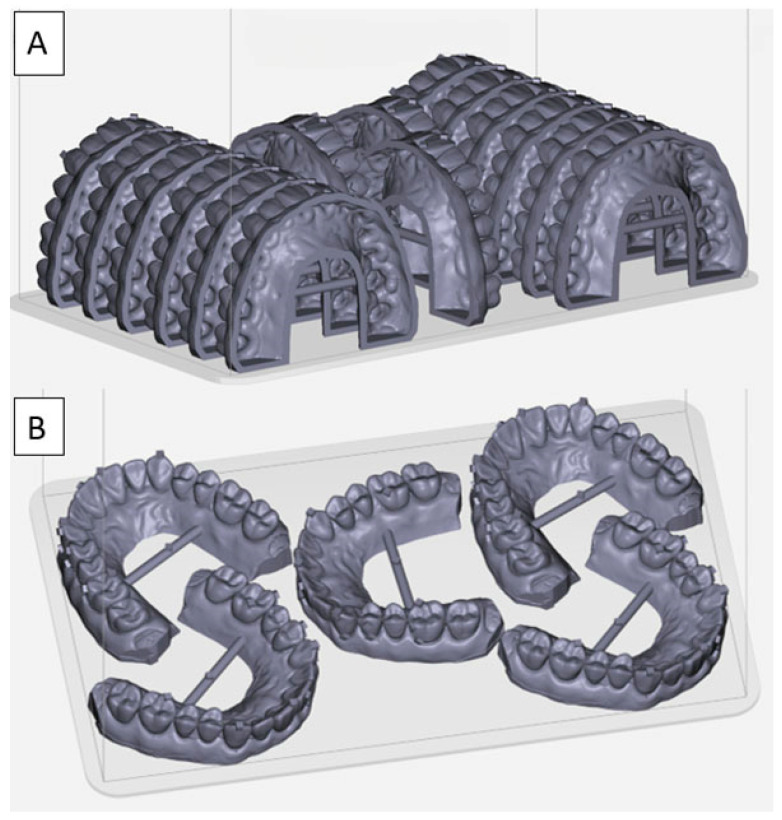
Digital models on the digital print platform. (**A**). Fifteen 90° shell models on the digital print platform. (**B**). Five 0° solid models on the digital print platform.

The current findings that almost all mean measurement values for all the models were clinically acceptable suggests that clinicians can utilize varying printing strategies as indicated by various clinical scenarios. For example, if a practitioner desired to print several models overnight and there is not a strict time restraint, then printing at 90° is advantageous, as more models can be fit onto the print platform. As shown in Figure 4A, fifteen shell models can be printed at a time. According to the printing software’s preview, the printing of this layout will take 100 min, and each model uses 9 mL of resin, which costs about $1.35 per model. Using solid models in an otherwise unchanged fifteen-model print job, the printing will take 129 min and require about 17 mL of resin per model at a cost of about $2.50/model. If a faster turnaround time is needed, then printing at 0° is preferred (Figure 4B). In this scenario, only five models fit on the platform, but the printing only takes 43 min to finish. A solid model is preferred for the 0° print angle due to the extra labor to create and fill the vent hole as described earlier, and the amount of resin and cost per model will remain at 17 mL of resin and $2.50 per model, respectively.

### 4.2. Study Limitations

The present study was performed with only one specific DLP printer to print the models. While the selected printer can be considered a standard for dental practices, the results do not necessarily apply to all DLP printers, nor do they apply to printers utilizing different 3D printing technologies, such as SLA, polyjet, or FDM. Further, in our study we rotated the position of the models on the platform to eliminate influence of the position. It cannot be ruled out that by this procedure a higher deviation from ideal model dimensions in a certain position was removed through averaging, however, our data suggest that there is no consistent clinically relevant inaccuracy in dimensions solely based on the model’s position on the printing platform.

As this and similar studies evaluated the results based on clinical significance, an additional limitation is that clinical acceptability has not been defined by official bodies such as the International Standards Organization (ISO), as it has been performed for instance for impression materials. Hence, the literature shows variations in the definition of acceptable clinical accuracy between studies ranging from 0.16 mm to 0.5 mm [10,12]. The current study considered differences greater than ±0.25 mm for any single tooth measurement and ±0.5 mm for cross arch measurements as clinically significant. These numbers relate to the amount of tooth movement per aligner tray as well as the PDL width. It should also be noted that the parameters for accuracy in this study are specific to orthodontic purposes and should not be applied to other dental purposes such as restorative applications, which do not allow dimensional deviations of more than ±0.1 mm [22,25].

## 5. Conclusions

Under the conditions and limitations of this study, orthodontic models can be 3D printed at varying angles and model body types while maintaining clinically acceptable accuracy. The print angles and model body types assessed in this study are those that would be most preferred in an in-house aligner workflow.

## Data Availability

The data presented in this study are available on reasonable request from the corresponding author.

## Supplementary Materials

The following supporting information can be downloaded at: https://www.mdpi.com/article/10.3390/biomimetics9040217/s1, Figure S1: Center of the reference cube (digital master model). Transparency with crosshairs laid over the computer screen with arms of crosshairs bisecting the four corners of the cube; Figure S2: Models mounted on the microscope platform: A. Solid-0° model mounted to view the occlusal surface. B. Solid-0° model mounted to view the anterior labial surfaces. C. Solid-0° model mounted to view the posterior right buccal surfaces; Figure S3: Center of the reference cube (printed model). Transparency with crosshairs laid over the microscope display monitor with arms of crosshairs bisecting the four corners of the cube; Table S1: Mean measurements of experimental models in mm (N = 10 models per model type and print angle). Mean measurements were calculated from 30 measurements (three measurements per site on each of the 10 models per model type and print angle); Table S2: Number of individual measurements (N = 10 measured models per print angle for each model type) outside the range of clinical acceptability. Each site was measured three times and averaged on each model for comparison with the master model. The canine height measured on the shell-0° model was outside the range of clinical acceptability seven out of 10 times. In summary, the 0° models were more often outside the range of clinical acceptability than the other angles, and the shell models more often than the solid models.
