# Supplementary material for: Effect of Model Body Type and Print Angle on the Accuracy of 3D-Printed Orthodontic Models
Source: Biomimetics (Basel). 2024 Apr 6;9(4):217. doi: 10.3390/biomimetics9040217 (PMC11048263; doi:10.3390/biomimetics9040217)
Supplement: Supplementary file 1 [file biomimetics-09-00217-s001.zip › supplementary materials captions.pdf]

Figure S1. Center of the reference cube (digital master model). Transparency with crosshairs laid over the computer screen with arms of crosshairs bisecting the four corners of the cube.

Figure S2. Models mounted on the microscope platform: A. Solid-0° model mounted to view the occlusal surface. B. Solid-0° model mounted to view the anterior labial surfaces. C. Solid-0° model mounted to view the posterior right buccal surfaces.

Figure S3. Center of the reference cube (printed model). Transparency with crosshairs laid over the microscope display monitor with arms of crosshairs bisecting the four corners of the cube.

Table S1. Mean measurements of experimental models in mm (N=10 models per model type and print angle). Mean measurements were calculated from 30 measurements (three measurements per site on each of the 10 models per model type and print angle).

Table S2. Number of individual measurements (N=10 measured models per print angle for each model type) outside the range of clinical acceptability. Each site was measured three times and averaged on each model for comparison with the master model. The canine height measured on the shell-0° model was 7 out of 10 times outside the range of clinical acceptability. In summary, the 0° models were more often outside the range of clinical acceptability than the other angles, and the shell models more often than the solid models.
