# Supplementary material for: Effect of Model Body Type and Print Angle on the Accuracy of 3D-Printed Orthodontic Models
Source: Biomimetics (Basel). 2024 Apr 6;9(4):217. doi: 10.3390/biomimetics9040217 (PMC11048263; doi:10.3390/biomimetics9040217)
Supplement: Supplementary file 1 [file biomimetics-09-00217-s001.zip › Table S1.pdf]

Table S1: Mean measurements of experimental models in mm (N=10 models per model type and print angle). Mean measurements were calculated from 30 measurements (three measurements per site on each of the 10 models per model type and print angle).

**Model type**	**Solid**			**Shell**		
**Print angle**	**0°**	**70°**	**90°**	**0°**	**70°**	**90°**
Incisor Height	-0.20 ± 0.03	-0.09 ± 0.04	-0.08 ± 0.06	-0.23 ± 0.03	-0.10 ± 0.04	-0.08 ± 0.06
Canine Height	-0.25 ± 0.03	-0.14 ± 0.05	-0.14 ± 0.04	-0.26 ± 0.03*	-0.19 ± 0.03	-0.14 ± 0.04
Premolar Height	-0.20 ± 0.02	-0.12 ± 0.03	-0.14 ± 0.04	-0.21 ± 0.03	-0.14 ± 0.06	-0.12 ± 0.04
Molar Height	-0.19 ± 0.02	-0.01 ± 0.04	-0.12 ± 0.05	-0.19 ± 0.05	-0.13 ± 0.05	-0.10 ± 0.02
Incisor Width	-0.04 ± 0.05	-0.01 ± 0.04	-0.03 ± 0.06	-0.03 ± 0.05	-0.02 ± 0.05	-0.09 ± 0.07
Canine Width	0.04 ± 0.05	0.01 ± 0.04	-0.02 ± 0.03	0.04 ± 0.04	0.01 ± 0.03	-0.05 ± 0.03
Premolar Width	-0.02 ± 0.03	-0.02 ± 0.04	-0.02 ± 0.03	-0.03 ± 0.04	-0.04 ± 0.03	-0.02 ± 0.03
Molar Width	0.02 ± 0.04	0.01 ± 0.03	0.03 ± 0.01	0.01 ± 0.05	-0.02 ± 0.02	-0.01 ± 0.02
Intermolar Distance	-0.15 ± 0.25	0.10 ± 0.23	0.13 ± 0.25	0.09 ± 0.27	0.37 ± 0.17	0.12 ± 0.30
Intercanine Distance	-0.04 ± 0.20	-0.07 ± 0.19	-0.07 ± 0.22	0.01 ± 0.24	0.07 ± 0.13	-0.21 ± 0.25
Arch Depth	-0.18 ± 0.08	-0.15 ± 0.05	-0.17 ± 0.06	-0.22 ± 0.12	-0.21 ± 0.06	-0.26 ± 0.04

* Only one mean measurement fell outside the selected range of clinical acceptability
