# Supplementary material for: Effect of Model Body Type and Print Angle on the Accuracy of 3D-Printed Orthodontic Models
Source: Biomimetics (Basel). 2024 Apr 6;9(4):217. doi: 10.3390/biomimetics9040217 (PMC11048263; doi:10.3390/biomimetics9040217)
Supplement: Supplementary file 1 [file biomimetics-09-00217-s001.zip › Table S2.pdf]

Table S2: Number of individual measurements (N=10 measured models per print angle for each model type) outside the range of clinical acceptability. Each site was measured three times and averaged on each model for comparison with the master model. The canine height measured on the shell-0° model was 7 out of 10 times outside the range of clinical acceptability. In summary, the 0° models were more often outside the range of clinical acceptability than the other angles, and the shell models more often than the solid models.

**Model type**		**Solid**			**Shell**	
**Print angle**	**0°**	**70°**	**90°**	**0°**	**70°**	**90°**
Incisor Height	1	0	0	0	0	0
Canine Height	2	0	0	7	0	0
Premolar Height	0	0	0	0	0	0
Molar Height	0	0	0	1	0	0
Incisor Width	0	0	0	0	0	0
Canine Width	0	0	0	0	0	0
Premolar Width	0	0	0	0	0	0
Molar Width	0	0	0	0	0	0
Intermolar Distance	1	0	0	0	1	2
Intercanine Distance	0	0	0	0	0	0
Arch Depth	0	0	0	0	0	0
Total	4	0	0	8	1	2
