# Supplementary figures and images for: Effect of Model Body Type and Print Angle on the Accuracy of 3D-Printed Orthodontic Models
Source: Biomimetics (Basel). 2024 Apr 6;9(4):217. doi: 10.3390/biomimetics9040217 (PMC11048263; doi:10.3390/biomimetics9040217)

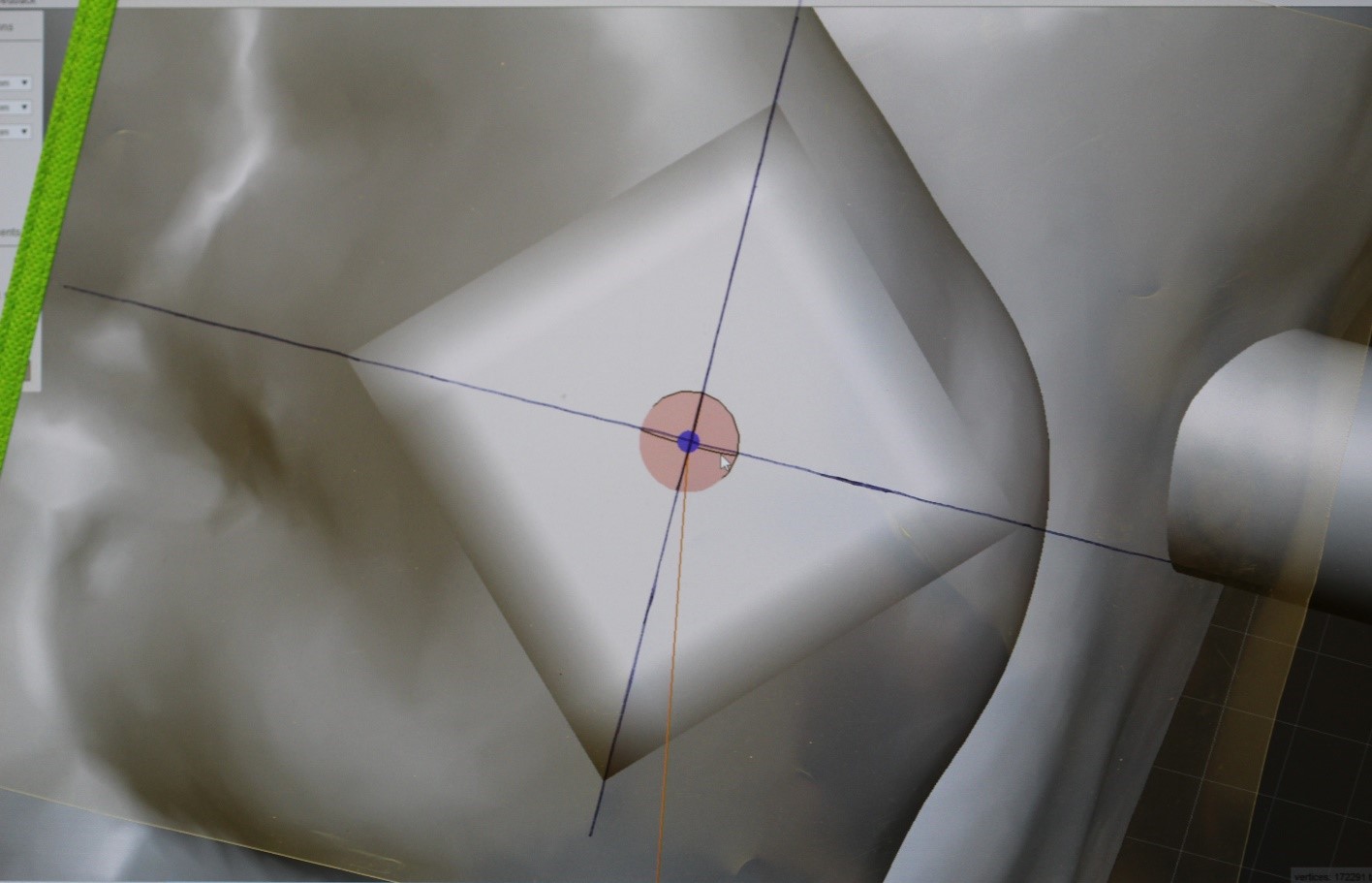

Supplement: Supplementary file 1 [file biomimetics-09-00217-s001.zip › Figure S1.jpg]

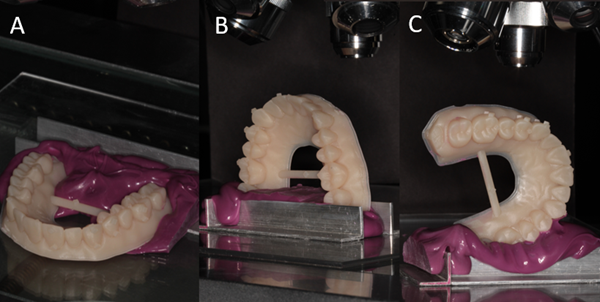

Supplement: Supplementary file 1 [file biomimetics-09-00217-s001.zip › Figure S2.png]

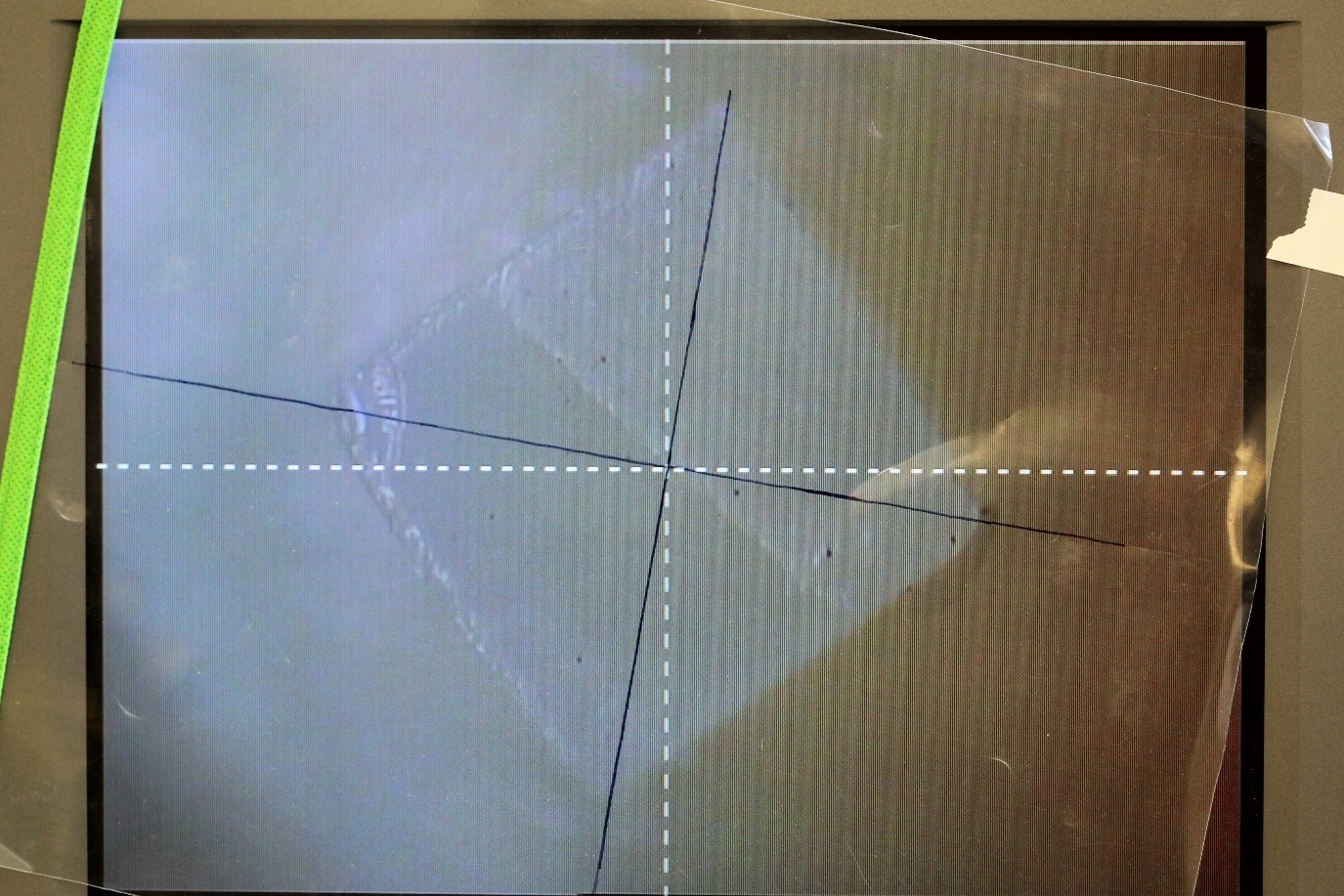

Supplement: Supplementary file 1 [file biomimetics-09-00217-s001.zip › Figure S3.jpg]
